# Durum Wheat Landraces from East and West Regions of the Mediterranean Basin Are Genetically Distinct for Yield Components and Phenology

**DOI:** 10.3389/fpls.2018.00080

**Published:** 2018-02-08

**Authors:** Jose M. Soriano, Dolors Villegas, Mark E. Sorrells, Conxita Royo

**Affiliations:** ^1^Sustainable Field Crops Programme, Institute for Food and Agricultural Research and Technology, Lleida, Spain; ^2^Department of Plant Breeding and Genetics, Cornell University, Ithaca, NY, United States

**Keywords:** association mapping, marker assisted selection, cycle length, yield components, Mediterranean basin

## Abstract

Genetic diversity of durum wheat landraces is a powerful tool for the introgression of new alleles of commercial interest in breeding programs. In a previous study, our team structured a collection of 172 durum wheat landraces from 21 Mediterranean countries in four genetic populations related to their geographical origin: east Mediterranean (17), east Balkan and Turkey (23), west Balkan and Egypt (25), and West Mediterranean (73), leaving 34 genotypes as admixed, and association mapping was carried out for important agronomic traits. Using a subset of this collection, the current study identified 23 marker alleles with a differential frequency in landraces from east and west regions of the Mediterranean Basin, which affected important agronomic traits. Eastern landraces had higher frequencies than the western ones of alleles increasing the number of spikes (wPt-5385 on chromosome 1B), grains per m^2^ (wPt-0841 on chromosome 7B), and grain filling duration (7 significant marker trait associations). Eastern landraces had higher frequencies of marker alleles located on chromosomes 4A, 5B, and 6B associated with reduced cycle length, and lighter grains than the western ones. Also for lower kernel weight, four marker alleles were located on chromosome 1A. Breeders may use the molecular markers identified in the current study for improving yield under specific Mediterranean environments.

## Introduction

Durum wheat (*Triticum turgidum* L. var. *durum*) is a self-pollinated tetraploid cereal and a traditional Mediterranean crop, with the Mediterranean Basin being the largest production area worldwide and North Africa the largest import market (Bonjean et al., 2016). Durum wheat is mainly used for the production of pasta and couscous, but also for a number of other semolina products such as frike, bourghul, and unleavened breads. In the Mediterranean Basin durum wheat is mainly cultivated under rainfed conditions where the precipitation is irregular across years and locations and along the plant growth cycle resulting in major yield variations.

Durum wheat originated and was domesticated in the Fertile Crescent (10,000 BP), and spread from the East to the West of the Mediterranean Basin (MacKey, 2005; Kabbaj et al., 2017) reaching the Iberian Peninsula around 7,000 years BP (Feldman, 2001). During the migration of wheat from the east to the west of the Mediterranean Basin, natural and human selection resulted in the establishment of local landraces, whose evolutionary dynamics are likely to have been strongly affected by environmental conditions, such as climatic variables, and soil properties (Mercer and Perales, 2010). These landraces were specifically adapted to their region of origin, representing a diversity of agro-ecological zones, and are considered to be the most important sources of biodiversity within the species (Nazco et al., 2012). Landraces were largely cultivated until the first decades of the twentieth century, being progressively abandoned from the early 1970s and replaced with improved, genetically uniform semi-dwarf cultivars as consequence of the Green Revolution (Ortiz et al., 2007). In addition, phenology trait-based breeding likely discarded important associated genetic traits. However, scientists believe that local landraces represent an important group of genetic resources for the improvement of commercially valuable traits (Lopes et al., 2015). Durum wheat Mediterranean landraces are considered as resources for contemporary agriculture to increase the genetic diversity of modern cultivated varieties and to improve their adaptation to regions affected by biotic and abiotic constraints.

Introgression of new alleles from locally adapted landraces into modern cultivars can be very useful when breeding for suboptimal environments such as those prevalent in the Mediterranean Basin. However, most landraces have still not been genetically nor agronomically characterized, although for their effective use in breeding, knowledge of molecular markers associated to alleles conferring resilience to the main constraints expected from climate change (erratic distribution of rainfall and temperature increases) become essential. Previous studies have demonstrated that the yield formation strategies of durum wheat are strongly affected by the environmental conditions. In warm and dry environments, as those prevalent in the east Mediterranean Basin, the number of spikes and grains per unit area are the most important yield components for yield formation, whereas in wetter and cooler environments, grain weight is more relevant due to both an extension of the grain filling period and water availability for photosynthesis (García del Moral et al., 2003; Moragues et al., 2006; Royo et al., 2006, 2014).

The efficient utilization of landraces in breeding programs requires understanding their genetic diversity and population structure. Royo et al. (2014) grouped 172 durum wheat landraces from 21 Mediterranean countries into four clusters based on the climatic data of their regions of origin. Later, Soriano et al. (2016), using molecular markers, structured the same collection in four genetic populations related to their geographic origin: eastern Mediterranean (EM), eastern Balkans and Turkey, western Balkans and Egypt, and western Mediterranean (WM). The genetic diversity found by Soriano et al. (2016) was lower within the eastern Mediterranean population, indicating that the diversity of durum wheat Mediterranean landraces increased during their migration to the western side of the Mediterranean Basin. Subsequently Soriano et al. (2017) reported an association mapping study that identified new marker trait associations (MTA) from the Mediterranean landraces for yield components, phenology, and biomass that have potential for improving modern durum varieties if the alleles prove to be superior in future studies.

Based on these results, the aim of this work was to identify MTAs with contrasting allelic effect for yield components and phenology traits in landraces from the east and the west of the Mediterranean Basin. Although Soriano et al. (2017) also reported association mapping for biomass traits, these were not included in the current study as the phenotypic means of landraces from the two regions were not statistically different (Soriano et al., 2016).

## Materials and methods

### Plant material

**Figure 1:**
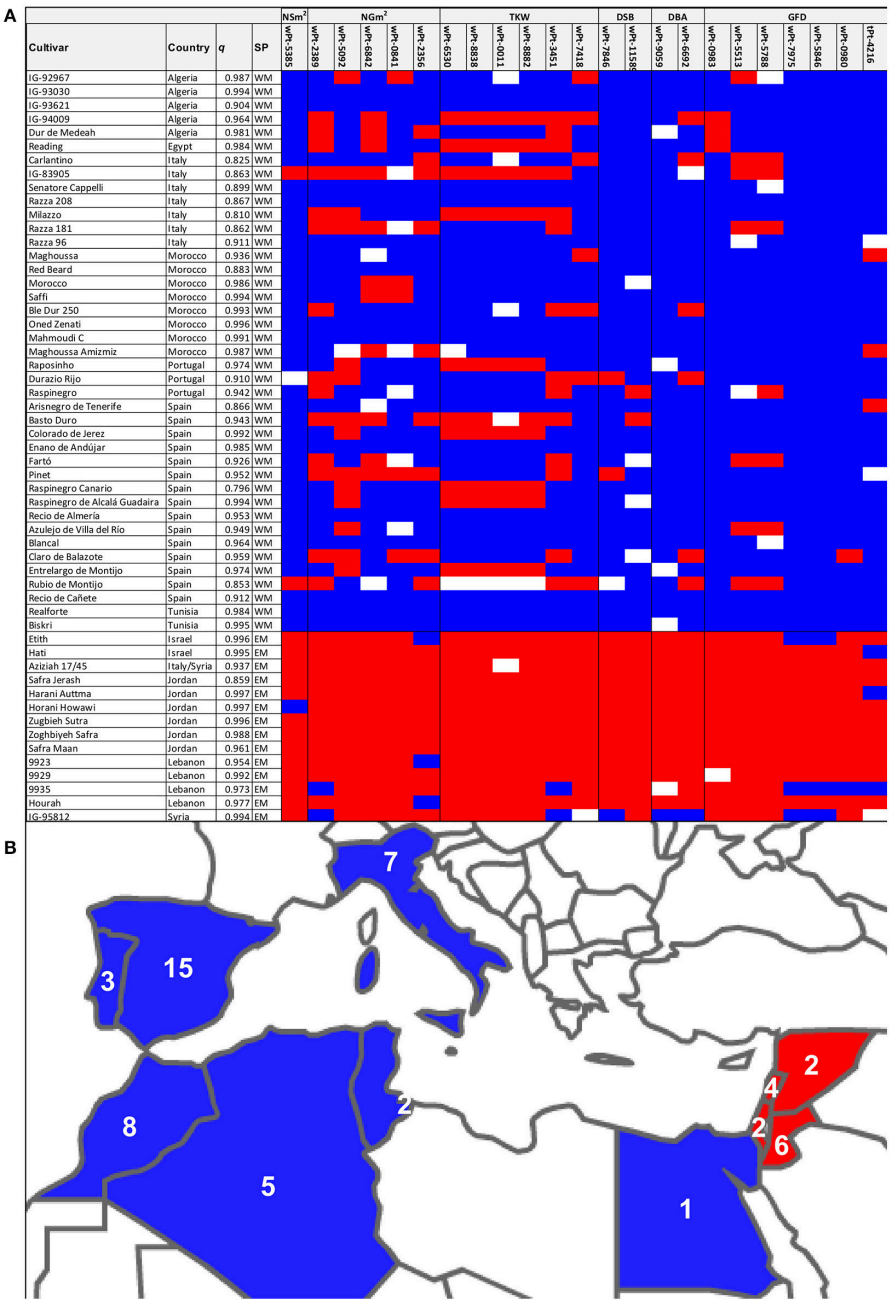
**(A)** Graphical genotyping for the selected eastern and western Mediterranean durum wheat landraces. Shown are the country of origin of the landraces, the membership coefficient (*q*) of the subpopulation (SP, EM = East Mediterranean, WM = West Mediterranean) of each landrace as reported by Soriano et al. (2016). Alleles present on each landrace are indicated by colors: Blue, west; red, east; white, not determined. **(B)** Map of the Mediterranean Basin highlighting the countries of origin of the landraces, indicating the number of landraces from each country.

Based on the population structure of durum wheat Mediterranean landraces revealed by Soriano et al. (2016), using a panel of 172 durum wheat landraces from the Mediterranean Basin (Royo et al., 2014; Soriano et al., 2016), the present work selected 14 genotypes from the eastern and 41 genotypes from the western Mediterranean populations with a membership coefficient *q* > 0.8 (Figure 1A). All but two cultivars, the Italian landrace “Aziziah 17/45” and the Egyptian “Reading,” were classified in the appropriate population according to their country of origin. “Aziziah 17/45” was included in the east Mediterranean population (*q* = 0.94). According to Scarascia Mugnozza (2005) although the cultivar was developed in Italy, it derived from early maturing pure line selected from Syro-Palestinian landraces. This would suggest that it could retain genetic background from the east of the Mediterranean Basin. While cultivar “Reading” with unknown pedigree, is classified by CRF (Centro de Recursos Fitogenéticos, INIA, Madrid) and reported by Nazco et al. (2012) as Egyptian, the molecular characterization carried out by Soriano et al. (2016) placed it in the west Mediterranean population (*q* = 0.98), which offers doubts about its geographical origin (Figure 1). Seeds were provided by Centro de Recursos Fitogenéticos (INIA-Spain), ICARDA Germplasm Bank and USDA Germplasm Bank and were sown and purified as described in Soriano et al. (2017).

### Plant phenotyping

Field experiments were carried out in two locations in Spain (Lleida, 41°40′N, 0°20′E, 260 m.a.s.l, in the north-east of Spain, referred as N, and Granada, 37°15′N, 3°46′W, 680 m.a.s.l, in the south of Spain, referred as S) during three harvesting seasons (2007, 2008, and 2009). Environments are further referred to as N or S followed by the year of crop harvest or by M, referred to as the calculated mean across years. Experiments consisted of an augmented design with three replicated checks (96 times) (cultivars “Claudio,” “Simeto,” and “Vitron”) and plots of 6 m^2^ (8 rows, 5 m long with a 0.15 m spacing). Sowing density was adjusted to 250 germinable seeds m^−2^ to avoid lodging. Meteorological data were recorded by weather stations placed in the experimental field (Table 1). Experiments were conducted under rainfed conditions, but irrigation was provided when necessary to allow seed germination. Weeds and diseases were controlled following standard practices at each site.

**Table 1:** Environmental details for the six field experiments.

Environment code	GR07	GR08	GR09	LL07	LL08	LL09
Site	Granada	Granada	Granada	Lleida	Lleida	Lleida
Year	2007	2008	2009	2007	2008	2009
Soil texture		Silty clay		Clay-loamy	Loamy	Sandy-clay-loamy
Sowing date (dd/mm/yy)	14/12/06	10/12/07	22/12/08	21/11/06	20/11/07	20/11/08
Harvest date (dd/mm/yy)	29/07/07	07/07/08	05/07/09	02/07/07	02/07/08	15/07/09
**Environmental data from sowing to harvest**						
Water input (mm)	320	345	337	208	258	237
Mean of daily minimum temperatures (°C)	7.5	7.0	7.1	6.1	6.5	6.3
Mean of daily maximum temperatures (°C)	22.8	22.1	21.9	17.4	18.6	18.3
Accumulated ET_0_ (mm)	803	673	614	533	755	623
Average daily relative air moisture (%)	55.9	58.6	60.5	73.4	88.7	70.5
Average daily solar radiation (MJ m^−2^ d^−1^)	19.4	18.4	17.8	14.5	14.9	15.9
Days sowing to anthesis modern cultivars	147	147	151	158	160	162
Days sowing to anthesis WM landraces	151	152	156	166	166	166
Days sowing to anthesis EM landraces	148	148	152	158	160	162

*Days from sowing to anthesis were determined by Soriano et al. (2016). WM, West Mediterranean subpopulation; EM, East Mediterranean subpopulation*.

The number of days from sowing to booting (DSB), number of days from booting to anthesis (DBA) and grain filling duration (GFD, days) were determined in each plot considering growth stages 45, 65, and 87, respectively, of the Zadoks scale (Zadoks et al., 1974). The number of spikes per square meter (NSm^2^) and the number of grains per square meter (NGm^2^) were measured at Zadoks GS87 from samples of the plants contained in a 1-m-long row from a central row of each plot. Thousand grain weight (TKW, g) was estimated as the mean weight of three sets of 100 g per plot.

### Graphical genotyping

From a total of 245 significant MTAs reported by Soriano et al. (2017), 126 corresponded to yield components (NSm^2^, NGm^2^, and TKW) and phenology (DSB, DBA, and GFD). Graphical genotyping (Young and Tanksley, 1989; van Eck et al., 2017) was used to select MTAs with different marker alleles for eastern and western Mediterranean subpopulations (Figure 1A). The marker data for each trait were loaded in Microsoft Excel, genotypes were shown in rows and the allele variant in columns (Figure 1A).

### Data analysis

Phenotypic data were fitted to a linear mixed model and restricted maximum likelihood was used to estimate the variance components and to produce the best linear unbiased estimates (BLUEs) for the phenotypic data using Genstat software v.18 (VSN International). Descriptive statistical data (mean, standard deviation, 95% confidence interval, skewness, and kurtosis) for the eastern and western populations defined by Soriano et al. (2016) and subpopulations defined in the present study, were calculated using JMP v8 statistical package (SAS Institute Inc, Cary, NC, USA) (Supplementary Table 1). Data were analyzed with the MIXED procedure of SAS statistical package (SAS Institute Inc, Cary, NC, USA) with the Kenward-Roger correction due to the unbalanced number of genotypes within subpopulations. Site, year, their interaction and genotype (within subpopulation) were considered random effects in the model (Smith et al., 2005). Mean comparisons for the phenotypic traits and for phenotypic means in the presence or absence of the markers were carried out using the Tukey-Kramer correction with the JMP v8 statistical package (SAS Institute Inc, Cary, NC, USA). The contribution of yield components to yield formation was assesses through stepwise regression analyses separately for eastern and western subpopulations. These analyses were conducted using the SAS statistical package (SAS Institute Inc, Cary, NC, USA) and considered yield as dependent variable and the main yield components as independent ones.

## Results

Descriptive statistics for the populations defined by Soriano et al. (2016) and the subpopulations used in the present study are shown in Supplementary Table 1 for yield, yield components, and phenology data. No statistically significant differences were found between both sets of landraces for the mean values using the Tukey-Kramer correction, thus validating the selection of genotypes within eastern and western subpopulations.

Mean values of phenotypic traits for the east and west subpopulations are shown in Table 2. No differences in grain yield were observed among subpopulations, probably due to compensation in yield components. In relation to yield components, landraces from the west region of the Mediterranean Basin had heavier grains, whereas yield for east Mediterranean landraces had increased numbers of spikes and grains per unit area. For phenology traits, eastern landraces flowered earlier and had a longer grain filling duration than the western ones (Table 2).

**Table 2:** Analysis of variance and mean values for yield components and phenology traits for landraces from east and west of the Mediterranean Basin.

**Environment**	**Yield**	**NSm^2^**	**NGm^2^**	**TKW**	**DSB**	**DBA**	**GFD**
East Mediterranean	312	441	7155	44	139	16	33
West Mediterranean	311	388	6236	50	143	17	31
Numerator D.F.	1	1	1	1	1	1	1
Denominator D.F.	53	53	53	53	53	53	53
*F* value	0.01	26.27	15.17	24.42	74.56	46.68	21.14
*P*-value	0.9377	<0.0001	0.0003	<0.0001	<0.0001	0.0002	<0.0001

*Data on first two rows are means across six environments. Yield, grain yield (g m^−2^); NSm^2^, number of spikes per m^2^; NGm^2^, number of grains per m^2^; TKW, thousand grain weight; DSB, days from sowing to booting; DBA, days from booting to anthesis; GFD, grain filling duration (days); DF, degrees of freedom*.

The contribution of the main yield components (NGm^2^ and TKW) to yield formation was assessed by regression analyses (Table 3). In landraces from the east of the Mediterranean Basin, the number of grains per unit area accounted by 84% of yield, whereas grain weight contributed by an additional 13%. On the other hand, for western Mediterranean landraces the contribution to yield of the number and weight of grains was much more balanced (54 and 44%, respectively).

**Table 3:** Regression equations for the contribution of main yield components to yield formation in eastern and western Mediterranean landraces.

**Environment**	**Regression equation**	**Partial *R*^2^**	**Model *R*^2^**	***P***
East Mediterranean	*Y* = −329.0 + 0.05 NGm^2^ + 7.06 TKW	NGm^2^: 0.84	0.84	<0.0001
		TKW: 0.13	0.97	<0.0001
West Mediterranean	*Y* = −305.3 + 0.05 NGm^2^ + 6.18 TKW	NGm^2^: 0.54	0.54	<0.0001
		TKW: 0.44	0.98	<0.0001

*NGm^2^, Number of grains per m^2^; TKW, thousand grain weight*.

Based on the mean differences among subpopulations, 23 MTAs with different effects in landraces from the east and the west of the Mediterranean Basin were found in five out of the six environments (N7, N8, N9, S7, and S8) and also for the mean values across environments (NM and SM) (Table 4). The allele associated to an increase in NSm^2^ was present in 93% of the East Mediterranean landraces and was strictly associated with this yield component. For NGm^2^, 76% of the landraces from the west Mediterranean had the allele associated with a decrease in number of grains per unit area and landraces from the east Mediterranean had the allele that increased them. Alleles with a high frequency in eastern landraces were associated with reduced grain weight whereas western landraces alleles were associated with heavier grains. The same correspondence was found for phenology traits. For days until anthesis (combined DSB and DBA) eastern genotypes had alleles associated with a shorter cycle (91%) and western alleles with a longer cycle (93%). For the GFD the effects were the opposite.

**Table 4:** Marker trait associations showing differential phenotypic effects on yield components and plant phenology in landraces from east and west of the Mediterranean Basin.

**Trait^a^**	**Marker**	**Chr**	**Position^b^**	**Env^c^**	**−log10(*P*)**	**Effect^d^**	**% (SP)^e^**	**Tukey's test^f^**		**F (*P-*value)**
								**E**	**W**	
NSm^2^	wPt-5385	1B	20.6	SM	3.3	14	93 (E)	449	384	28.0 (<0.0001)
NGm^2^	wPt-0841	7B	116.3	S8	3.0	647	100 (E)	7005	6150	14.4 (0.0004)
NGm^2^	wPt-2356	7B	160.7	N7/NM	4.3/3.4	373/262	79 (E)	6904	6222	9.3 (0.0035)
NGm^2^	wPt-5092	7A	2.2	N8	3.2	326	63 (W)	6607	6317	1.5 (0.22)
NGm^2^	wPt-2389	1B	42.9	N7	3.1	−355	66 (W)	6669	6292	2.7 (0.10)
NGm^2^	wPt-6842	7A	138.6	N7	3.2	−384	66 (W)	6767	6204	6.0 (0.018)
TKW	wPt-7418	5B	U	N8	3.7	−1.9	93 (E)	45	51	21.5 (<0.0001)
TKW	wPt-0011	1A	U	SM	3.1	−1.4	93 (E)	45	51	33.3 (<0.0001)
TKW	wPt-8882	1A	U	SM	3.7	−1.4	100 (E)	45	51	29.7 (<0.0001)
TKW	wPt-6530	1A	U	SM	3.4	−1.3	100 (E)	45	51	29.9 (<0.0001)
TKW	wPt-8838	1A	U	SM	3.2	−1.3	100 (E)	45	51	29.7 (<0.0001)
TKW	wPt-3451	1B	43.9	NM	3.0	1.5	66 (W)	45	51	35.1 (<0.0001)
DSB	wPt-7846	6B	60.3	N8/NM	4/3.5	−1.4/−1.2	93 (E)	140	143	31.9 (<0.0001)
DSB	wPt-11589	6B	83.6	S7/SM	4.6	−1/−1	86 (E)	140	143	31.8 (<0.0001)
DBA	wPt-9059	4A	136.8	N7	3.9	−0.6	86 (E)	16	17	15.8 (0.0002)
DBA	wPt-6692	5B	25.6	N8/NM	3.1/3.2	−0.5/−0.4	93 (E)	16	17	15.0 (0.0003)
GFD	wPt-0980	7B	117.6	N8	3.1	1.1	93 (E)	33	31	16.3 (0.0002)
GFD	wPt-7975	7B	3.6	NM	3.6	1.0	79 (E)	33	31	16.7 (0.0002)
GFD	wPt-5846	7B	3.6	NM	3.6	1.0	79 (E)	33	31	16.7 (0.0002)
GFD	wPt-0983	1B	−7.9	S8	3.5	0.8	93 (E)	33	31	11.8 (0.0012)
GFD	wPt-5513	2B	45.2	N8	3.2	0.8	100 (E)	32	31	4.5 (0.038)
GFD	wPt-5788	2B	45.3	N8	3.0	0.8	100 (E)	32	31	5.0 (0.029)
GFD	tPt-4216	N	U	N9	3.8	0.7	71 (E)	33	31	15.4 (0.0003)

a *NSm^2^, number of spikes per m^2^; NGm^2^, number of grains per m^2^; TKW, thousand grain weight (g); DSB, days from sowing to booting; DBA, days from booting to anthesis; GFD, grain filling duration (days)*.

b *U, Unmapped*.

c *Environments: N7, North 2007; N8, North 2008; N9, North 2009; NM, North mean; S7, South 2007; S8, South 2008; SM, South mean*.

d *Allele effect calculated with GenStat v18. Units of the effect are in accordance with those reported for each trait*.

e *Frequency of the genotypes carrying the allele conferring the show effect. In parenthesis, E, east Mediterranean; W, west Mediterranean*.

f *Mean comparisons among the presence of the east (E)/west (W) allele for each one of the analyzed traits*.

**Figure 2:**
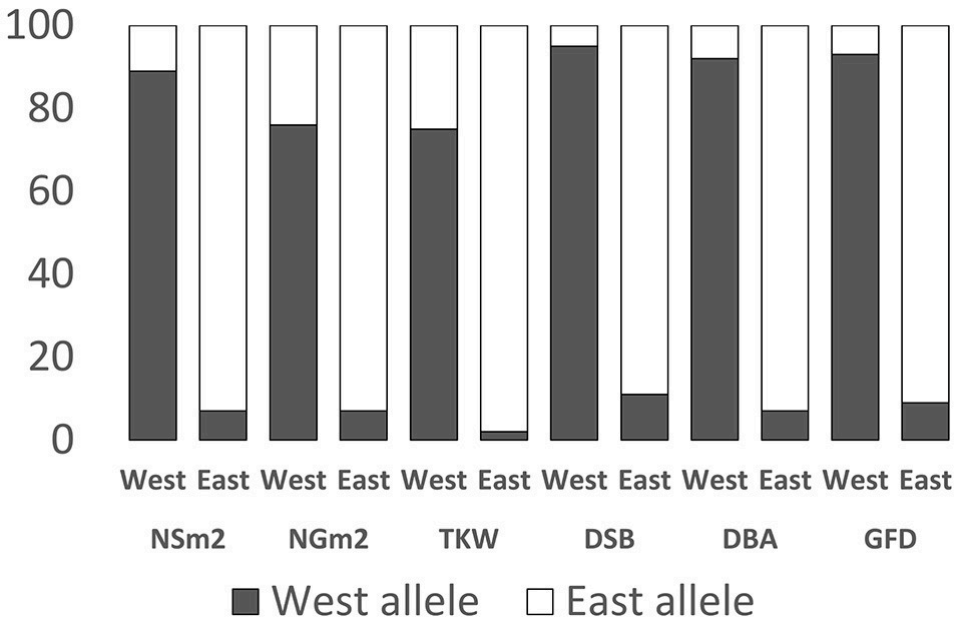
Marker allele frequencies specific to landraces from east and west of the Mediterranean Basin for the analyzed traits. All the significant markers shown in Table 2 and Figure 1 are included. NSm^2^, number of spikes per m^2^; NGm^2^, number of grains per m^2^; TKW, thousand grain weight (g); DSB, days from sowing to booting; DBA, days from booting to anthesis; GFD, grain filling duration (days).

Figure 2 shows the frequency of the alleles in landraces from countries located east or west of the Mediterranean Basin for each trait. Marker allele frequencies specific to the western landraces range from 75% for TKW to 95% for DSB with a mean of 87%, whereas marker allele frequencies specific to the eastern landraces range from 89% for DSB to 98% for TKW with a mean of 93%.

To associate allele frequencies of eastern or western landraces with agronomic performance of genotypes, the mean values were compared for each MTA using the Tukey's test for each marker. Only two MTAs for NGm^2^ (wPt-2389 and wPt-5092) were not statistically significant at *P* < 0.05 (Table 4).

## Discussion

Domestication and breeding resulted in a gradual loss of genetic variability. The use of wheat landraces is considered a good approach to recover and to broaden allelic variation of traits of interest (Lopes et al., 2015). The availability of landraces for breeding programs may be of particular interest when breeding for suboptimal environments such as the Mediterranean Basin. Although phenology traits such as early heading or flowering are present mainly in modern cultivars, Soriano et al. (2016) found no significant differences for these traits among modern durum wheat cultivars and east Mediterranean landraces. Mediterranean durum wheat landraces are an important group of genetic resources because of their huge genetic diversity and specific adaptation to local environments (Nazco et al., 2012), and resistance to pests and pathogens (DuToit, 1989; Kyzeridis et al., 1995; Talas et al., 2011; Valdez et al., 2012). They are also considered to be the most important sources of biodiversity within the species (Nazco et al., 2012). Collections of landraces and modern cultivars are being phenotyped at CIMMYT to unravel the genetic basis of drought and heat tolerance (Lopes et al., 2015). Yield component traits as TKW are being also evaluated. As reported by Lopes et al. (2012), the increase in TKW was associated with rises in grain yield in environments with low or medium yield gains. Similarly, Soriano et al. (2016) found heavier grains in western Mediterranean landraces than in modern cultivars.

The population structure of the collection of durum wheat Mediterranean landraces from which the present genotypes were derived showed a clear genetic classification that matched with the geographical origin of the landraces (Soriano et al., 2016). Based on this genetic analysis and the contrasting agronomic performance of landraces from different climatic regions within the Mediterranean Basin reported by Royo et al. (2014), landraces from the western and eastern regions of the Mediterranean Basin were selected for the current study according to the membership coefficient given by STRUCTURE software (*q*) (Pritchard et al., 2000).

Previous studies have revealed that the relative contribution of yield components to yield formation in durum wheat is strongly affected by the meteorological conditions prevalent during the crop growth, principally temperature and water availability (García del Moral et al., 2003; Moragues et al., 2006; Royo et al., 2006, 2014). Based on this assumption, the current study aimed to elucidate whether the frequency of allelic variants associated to the main yield components differed in landraces from east and west of the Mediterranean Basin. The Koeppen's climatic classification establishes contrasting climatic characteristics for these two sides of the Mediterranean Basin (Leemans and Cramer, 1991). The analysis of long-term climatic data has demonstrated that the eastern countries Syria, Lebanon, Israel and Jordan are the warmest and driest within the Mediterranean region (Royo et al., 2014).

Yield components are sequentially formed in wheat, with the latter-developing components being under the influence of earlier-developing ones (García del Moral et al., 1991; Dofing and Knight, 1992). The potential number of spikes per unit area is defined during the vegetative phase and it is strongly affected by water availability (García del Moral et al., 1991; Simane et al., 1993) and differences in soil conditions (i.e., soil fertility and nitrogen availability). The fact that eastern landraces had larger numbers of spikes per unit area than those from the west Mediterranean countries, may be interpreted as an evolutionary adaptive mechanism to compensate for the negative effect of water scarcity on the formation of spikes. Similarly, the larger number of grains per unit area, whose potential is determined before booting (Isidro et al., 2011), may be a means to compensate for the degeneration of florets occurring from booting to flowering (Isidro et al., 2011) and the subsequent reduction in grain setting caused by heat and drought stress (Barber et al., 2017). The high frequency of alleles conferring a high number of spikes and grains in landraces collected in the four eastern countries (93% in both cases) may be biologically interpreted as a mechanism for achieving higher grain yield. The reason is that the grain yield relies on the production of a large number of reproductive organs and a delay in senescence when plants are grown in harsh environments (Albacete et al., 2014). The results obtained in this study fully match with previous studies (Moragues et al., 2006; Royo et al., 2014) showing the importance of the number of spikes and grains on the yield formation strategy of durum wheat grown in warm and dry areas.

Grain weight is the last yield component formed and is usually negatively correlated with grain number (Sadras, 2007). Therefore, it is not surprising that landraces collected in eastern Mediterranean countries had a high frequency (98%) of alleles associated with low grain weight. The current study demonstrated that the landraces from the east had lighter grains than those from the west of the Mediterranean Basin, in agreement with the low contribution of grain weight to yield as demonstrated by regression analysis. Two likely physiological reasons may explain it. The first could be a constraint in the photosynthates available for grain filling due to the decrease caused in current photosynthesis by unfavorable environmental conditions after flowering (Royo et al., 1999, 2017). The second could derive from the distribution of the available carbohydrates on a large number of grains to be filled (sink size), thus resulting in lighter grains. On the other hand, landraces from the west of the Mediterranean Basin had a high frequency of alleles mostly conferring opposite characteristics to those of the eastern area: low number of grains per unit area (76%) and high kernel weight (75%). The results of the regression analyses showing a much more balanced contribution of grain number and weight to yield in western landraces than in eastern ones are in agreement with the allelic frequencies obtained. These findings are consistent with previous studies showing a higher grain filling rate in landraces from cold and wet areas, associated with heavier grains in durum wheat (Motzo et al., 1996; Royo et al., 2014). It has been reported that grain weight in wheat is much more constrained under terminal drought conditions than in cold and wet environments (Royo et al., 2000). The selection for heavier grains during the spreading of durum wheat across the Mediterranean Basin may also have contributed (Peng et al., 2011).

The short time to booting of landraces from the East of the Mediterranean Basin may be also interpreted as an evolutionary adaptive mechanism to escape from the high temperatures, radiation, potential evapotranspiration and water scarcity usual in this area after flowering (Royo et al., 2014). Early flowering is one of the most common physiological mechanisms for escaping drought. The presence in eastern genotypes of a high frequency of alleles reducing time to flowering (89%) is in agreement with previous studies showing that drought stress and high temperature in the collecting site of durum wheat landraces reduced cycle length (Annicchiarico et al., 1995). Solar radiation has been reported to be one of the climatic variables most affecting the cycle length of landraces, with those from areas with high solar radiation having the shortest one (Royo et al., 2014).

The results of previous studies in wheat that associated early flowering with longer grain filling duration (Tewolde et al., 2006; Royo et al., 2014) are supported by the findings of the current study as 91% of the eastern landraces carried the allele increasing grain filling duration and 93% of the western genotypes carried the allele reducing it. A previous study revealed that eastern Mediterranean landraces had a slower grain filling rate than the western ones (Royo et al., 2014). The results of the current research showed that despite having a longer grain filling duration the grains formed were lighter, which is in agreement with the high frequency in eastern landraces of alleles associated to low grain weight found in the current study (98%).

The results obtained in this study support the theory that during the migration of wheat from the east to the west of the Mediterranean Basin, it developed adaptive mechanisms to the different conditions found in the new areas, which led to new strategies for yield formation. The frequency of alleles associated to the rise or decrease of certain yield components in regions with contrasting environmental conditions strongly suggest that the different yield formation strategies of landraces from the east and the west of the Mediterranean Basin is based on genetic differences. Moreover, our results give clues for the improvement of varieties better adapted to specific environmental conditions. The use of specific alleles affecting grain number or weight, as well as grain filling duration, will allow fine-tuning the most favorable allelic combinations for increasing yield in target regions across the Mediterranean Basin especially in regards to predicted climate change scenarios.

## Author contributions

CR: Designed the experiments; CR and DV: Assembled and purified the germplasm collection; JS, DV, MS, and CR: Performed field evaluations and/or data analyses; JS, MS, and CR: Conceived the manuscript; JS and CR: Wrote the manuscript. JS, DV, MS, and CR: Edited and provided critical review of the manuscript; JS, DV, MS, and CR: Read and approved the final manuscript.

### Conflict of interest statement

The authors declare that the research was conducted in the absence of any commercial or financial relationships that could be construed as a potential conflict of interest.

## Abbreviations

DSB: Days from sowing to booting
DBA: days from booting to anthesis
EM: east Mediterranean
GFD: grain filling duration
MTA: marker trait association
NSm^2^: number of spikes per m^2^
NGm^2^: number of grains per m^2^
TKW: thousand grain weight
WM: west Mediterranean.
