# Supplementary material for: Durum Wheat Landraces from East and West Regions of the Mediterranean Basin Are Genetically Distinct for Yield Components and Phenology
Source: Front Plant Sci. 2018 Feb 8;9:80. doi: 10.3389/fpls.2018.00080 (PMC5809869; doi:10.3389/fpls.2018.00080)
Supplement: Supplementary file 1 [file Table1.docx]

Supplementary Material

Durum wheat landraces from East and West Regions of the Mediterranean Basin are genetically distinct for yield components and phenology

**Jose Miguel Soriano, Dolors Villegas, Mark Earl Sorrells, Conxita Royo^*^**.

*** Correspondence:** Conxita Royo: conxita.royo@irta.cat

**Supplementary Table 1** Descriptive statistics for the western (W) and eastern (E) landrace subpopulations reported by Soriano et al. (2016) and for the subpopulations selected within each of them for the present study. E-W, WM and EM correspond to the subpopulations handled in Soriano et al. (2016), while Sub E-W, Sub WM and Sub EM correspond to the selected genotypes within each subpopulation used in the present study. F and *P*-value correspond to the mean differences among the entire subpopulations and the selected genotypes using the Tukey-Kramer correction.

|  |  | Yield | NSm^2^ | NGm^2^ | TKW | DSB | DBA | GFD |
| --- | --- | --- | --- | --- | --- | --- | --- | --- |
| E-W | Mean | 312 | 407 | 6594 | 47.7 | 142 | 16 | 31 |
|  | SD | 36 | 41 | 840 | 4.6 | 2.4 | 1.0 | 1.2 |
|  | Lower CI | 305 | 398 | 6419 | 46.7 | 141 | 16 | 31 |
|  | Upper CI | 320 | 415 | 6769 | 48.6 | 143 | 16 | 32 |
|  | Skewness | 0.046 | 0.533 | 0.235 | 0.010 | -0.444 | -0.014 | 0.283 |
|  | Kurtosi | -0.645 | -0.152 | 0.207 | -0.077 | 0.257 | 0.203 | 0.678 |
| Sub E-W | Mean | 312 | 402 | 6470 | 48.5 | 142 | 16 | 31 |
|  | SD | 38 | 40 | 856 | 4.7 | 2.1 | 0.9 | 1.3 |
|  | Lower CI | 302 | 391 | 6238 | 47.2 | 141 | 16.3 | 31 |
|  | Upper CI | 322 | 413 | 6701 | 49.8 | 143 | 16.7 | 32 |
|  | Skewness | -0.055 | 0.566 | -0.072 | 0.050 | -0.1758 | -0.113 | 0.119 |
|  | Kurtosi | -0.660 | -0.258 | 0.305 | -0.430 | -0.268 | 0.249 | 0.871 |
|  | F | 0.0084 | 0.4627 | 0.7355 | 1.1036 | 0.1129 | 1.1298 | 0.0234 |
|  | *P*-value | 0.9272 | 0.4975 | 0.3925 | 0.2952 | 0.7374 | 0.2896 | 0.8787 |
| WM | Mean | 312 | 398 | 6462 | 48.6 | 143 | 16 | 31 |
|  | SD | 37 | 39 | 832 | 4.6 | 1.8 | 1.1 | 1.0 |
|  | Lower CI | 304 | 389 | 6266 | 47.5 | 142 | 16 | 31. |
|  | Upper CI | 321 | 407 | 6658 | 49.7 | 143 | 17 | 31 |
|  | Skewness | 0.156 | 0.798 | 0.470 | -0.344 | -0.073 | -0.175 | -0.194 |
|  | Kurtosi | -0.765 | 0.666 | 0.872 | 0.328 | 1.255 | 0.216 | 0.611 |
| Sub-WM | Mean | 311 | 388 | 6236 | 50.1 | 143 | 16 | 31 |
|  | SD | 40 | 33 | 773 | 4.4 | 1.4 | 0.8 | 1.1 |
|  | Lower CI | 298 | 378 | 5992 | 48.6 | 142 | 16 | 31 |
|  | Upper CI | 324 | 399 | 6480 | 51.4 | 143 | 17 | 31 |
|  | Skewness | -0.031 | 0.881 | -0.247 | -0.505 | 0.424 | -0.191 | -0.59 |
|  | Kurtosi | -0.824 | 1.262 | 0.566 | 0.829 | 0.547 | 0.785 | -0.400 |
|  | F | 0.0301 | 1.7338 | 2.0302 | 2.5673 | 0.0648 | 2.6638 | 0.3627 |
|  | *P*-value | 0.8625 | 0.1906 | 0.157 | 0.1119 | 0.7995 | 0.1055 | 0.5482 |
| EM | Mean | 313 | 440 | 7093 | 44.1 | 139 | 15 | 32 |
|  | SD | 35 | 34 | 682 | 2.2 | 1.6 | 0.7 | 1.2 |
|  | Lower CI | 296 | 423 | 6764 | 43.0 | 138 | 15 | 32 |
|  | Upper CI | 330 | 456 | 7422 | 45.2 | 140 | 16 | 33 |
|  | Skewness | -0.503 | 0.343 | -0.339 | -0.522 | -0.309 | -0.252 | -0.093 |
|  | Kurtosi | 0.296 | -1.183 | -0.684 | 0.708 | 0.007 | -0.745 | 0.532 |
| Sub-EM | Mean | 314 | 441 | 7155 | 43.9 | 139 | 15 | 32 |
|  | SD | 30 | 33 | 729 | 1.8 | 1.0 | 0.7 | 1.1 |
|  | Lower CI | 297 | 422 | 6734 | 42.9 | 138 | 15 | 32 |
|  | Upper CI | 331 | 461 | 7576 | 45.0 | 140 | 16 | 33 |
|  | Skewness | 0.005 | 0.164 | 0.139 | -0.067 | -0.376 | -0.291 | 0.950 |
|  | Kurtosi | -0.121 | -1.525 | -0.579 | 0.722 | -0.125 | 1.305 | 0.917 |
|  | F | 0.0158 | 0.0208 | 0.0629 | 0.0169 | 0.8371 | 0.2584 | 0.0071 |
|  | *P*-value | 0.9007 | 0.8864 | 0.8037 | 0.8974 | 0.3673 | 0.6148 | 0.9336 |

Yield, grain yield (g m^-2^); NSm^2^, number of spikes per m^2^; NGm^2^, number of grains per m^2^; TKW, thousand grain weight; DSB, days from sowing to booting; DBA, days from booting to anthesis; GFD, grain filling duration (days).
